# NMR-based evaluation of the metabolic profile and response to dichloroacetate of human prostate cancer cells

**DOI:** 10.1002/nbm.3101

**Published:** 2014-03-17

**Authors:** Mithun Kailavasan, Ishtiaq Rehman, Steven Reynolds, Adriana Bucur, Gillian Tozer, Martyn Paley

**Affiliations:** a Department of Cardiovascular Science, The Medical School, University of SheffieldSheffield, UK; b Department of Oncology, Sheffield CRUK/YCR Cancer Research Centre, The Medical School, University of SheffieldBeech Hill Road, Sheffield, UK

**Keywords:** prostate cancer, dichloroacetate, bioreactor, zymography, lactate dehydrogenase, prostate-specific antigen, metabolic biomarkers, NMR

## Abstract

The aim of this study was to evaluate the metabolic profile of human prostate cancer cells that have different metastatic potential and to determine their response to dichloroacetate (DCA) using NMR technology. Two isogenic human prostate cancer cell lines, differing in their metastatic potential [LNCaP (poorly metastatic) and LNCaP-LN3 (highly metastatic)], were studied. Metabolite ratios from NMR spectral integrals acquired at a field strength of 9.4 T using a 5-mm broadband probe with an NMR-compatible bioreactor were compared in the presence and absence of the pyruvate dehydrogenase kinase inhibitor DCA. Lactate dehydrogenase (LDH) isoenzymes were assessed by zymography. Following the treatment of cells with 50 mm DCA, there was a significant reduction in the lactate/choline, lactate/creatine, lactate/alanine and the combined lactate/(choline + creatine + alanine) ratios in LNCaP-LN3 cells relative to LNCaP cells. No significant changes in metabolite ratios were found in LNCaP cells following DCA treatment. As expected, LDH zymography assays showed an absence of the LDH-B subunit in LNCaP-LN3 cells, whereas both LDH-A and LDH-B subunits were present in LNCaP cells. DCA was shown to significantly modify the metabolite ratios in highly metastatic LNCaP-LN3 cells, but not in poorly metastatic LNCaP cells. This effect was probably related to the absence of the LDH-B subunit in LNCaP-LN3 cells, and could have a bearing on cancer treatment with DCA and related compounds. © 2014 The Authors. *NMR in Biomedicine* published by John Wiley & Sons, Ltd.

## INTRODUCTION

Worldwide, prostate cancer is the second most common cancer diagnosis 1. In 2012, the American Cancer Society estimated that around 241 740 American men were diagnosed with prostate cancer and 28 170 men died from the disease 1,2. The vast majority of cancer-related deaths were caused by progression of the disease from being confined to the organ to disseminated metastatic disease 3. As a result of the heterogeneity of the biological behaviour of prostate cancer, a major challenge facing clinicians in the management of patients is distinguishing between the subset of patients in whom the disease will progress to become a lethal metastatic disease and the majority of patients in whom the tumour will remain organ confined and relatively indolent.

Current methods of detection and diagnosis of prostate cancer are based on a triad of serum prostate-specific antigen (PSA) measurements, digital rectal examination (DRE) and histological assessment of trans-rectal ultrasound (TRUS)-guided needle biopsy material 4,5. Although PSA is a US Food and Drug Administration-approved biomarker, and the most widely used biomarker for prostate cancer detection, its use is associated with a high degree of false-positive and false-negative test results 6. Therefore, PSA testing has led to an over-diagnosis and over-treatment of otherwise clinically insignificant disease 7. Thus, there is an urgent need for additional biomarkers that can improve the diagnostic specificity of PSA, and reliably predict the likelihood of disease progression.

**Figure 1:**
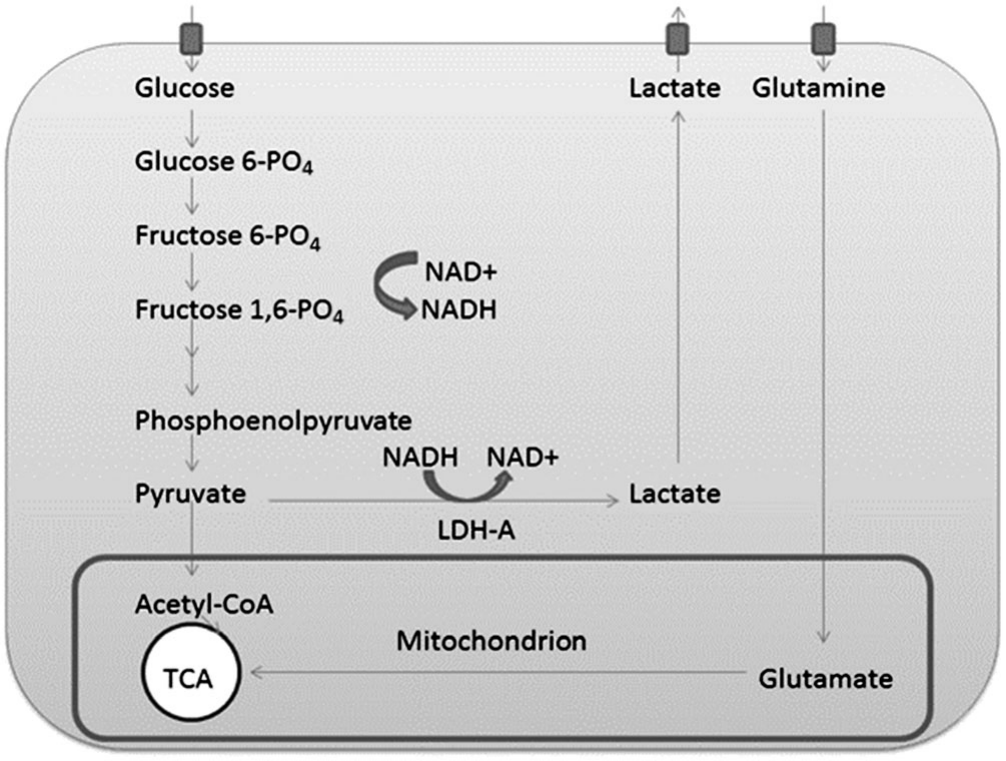
Simplified diagram containing the cellular anaerobic glycolytic pathway and tricarboxylic acid (TCA) cycle: note the regeneration of NAD^+^ through the glycolysis process. Some of the steps between fructose 1,6-phosphate (fructose 1,6-PO_4_) and phosphoenolpyruvate have been omitted for clarity.

In the presence of oxygen, normal cells derive most of their energy in the form of ATP from glycolysis, coupled with mitochondrial oxidative phosphorylation, in a process that yields a total of 36 ATP molecules per molecule of glucose consumed, in addition to CO_2_ and water 8. However, when the supply of oxygen is limited, pyruvate is converted to lactate by a reaction catalysed by lactate dehydrogenase (LDH) (Fig. 1). LDH is a tetrameric enzyme, consisting of different combinations of two subunits (LDH-A and LDH-B, encoded by two different genes) to form five different isoenzymes LDH-1–LDH-5: LDH-B4 (LDH-1); LDH-B3:A1 (LDH-2); LDH-B2:A2 (LDH-3), LDH-B1:A3 (LDH-4) and LDH-A4 (LDH-5) subunits 9,10.

In contrast with normal cells, cancer cells derive a higher proportion of their energy from lactic acid fermentation, even in the presence of oxygen, despite the fact that it is a highly inefficient process. A high rate of aerobic glycolysis, followed by lactic acid fermentation in the cytosol, was first described in cancer cells by Otto Warburg in the 1920s, and is now known as the Warburg effect 11. Conversion of pyruvate to lactate regenerates the oxidised form of nicotinamide adenine dinucleotide (NAD^+^), necessary as a co-factor for continued glycolysis and associated anabolic pathways, which are needed for tumour cell proliferation. Thus, the altered metabolism seen in cancer cells is thought to confer a survival advantage to the cells 12.

A number of other metabolic changes have been reported in cancer cells. For example, the MYC gene that regulates mitochondrial glutamine metabolism is frequently dysregulated in human cancers 13. Thus, cancer cells exhibit increased glutamine metabolism, and the resulting products are used as precursors for the synthesis of other amino acids, such as proline, arginine and ornithine 14. Other studies have estimated that around 60% of glutamine in cancer cells is used to promote lactate and alanine production 12. Cancer cells have also been shown to have elevated *de novo* fatty acid biosynthesis, which has been associated with evasion from cellular apoptosis 12. However, certain cancer cells may display signs of increased fatty acid oxidation, as it can provide an alternative energy source 15,16. There is good evidence that increased lactate metabolism is associated with a higher rate of metastasis formation, and that the targeting of lactate production with drugs, such as dichloroacetate (DCA) (see below), could be effective against metastases 17.

Collectively, a number of metabolic changes have been associated with neoplastic transformation, in particular increased intracellular glucose metabolism, increased glycolytic enzyme activities (including LDH activity), glutaminolytic activity and *de novo* fatty acid synthesis 18, as well as increased choline, phosphocholine and alanine 19–21. Pyruvate can be converted into alanine via the transaminase reaction, which is in rapid equilibrium. The lactate/alanine (Lac/Ala) ratio is associated with a cellular redox state because the conversion of pyruvate to lactate and alanine is coupled with NAD^+^ and NADH reactions 22. Thus, the Lac/Ala ratio reflects the NADH/NAD^+^ equilibrium 23.

DCA is a small molecule of 151 Da with high bioavailability that has undergone clinical trials for the treatment of a number of cancer types. For instance, a published Phase 1 clinical trial studied the effect of DCA in patients suffering from glioblastoma, and there have been calls for a Phase 2 trial 24. *In vitro*, DCA has been shown to inhibit pyruvate dehydrogenase kinase (PDK), including the PDKII isoform. PDKII is one of four isoforms of PDK, that is expressed in most tissues and has the highest sensitivity to DCA 24. An inhibition of PDK is associated with an increase in pyruvate dehydrogenase activity, and the consequences of this inhibition are thought to be an increase in the flux of pyruvate into the mitochondria of the cell, thereby promoting oxidative phosphorylation over lactate production and restoring a ‘normal’ metabolic state in cancer cells. However, although these *in vitro* and *in vivo* studies have suggested a beneficial role for DCA in the treatment of human cancers 25–27, side effects from toxicity have hindered development. Studies in mice have demonstrated neurotoxicity and, in high doses, DCA was found to be carcinogenic 26. Currently, DCA remains a useful prototype PDK inhibitor for use in preclinical studies until more specific agents become available. Tumour response to DCA is highly variable, depending on the tumour model used, thus highlighting the need for predictive biomarkers that can predict the therapeutic response to PDK inhibitors.

The aim of the present study was to employ NMR techniques to characterise the metabolic profile of two isogeneic human prostate cancer cell lines, which differ in their *in vivo* metastatic potentials [LNCaP (poorly metastatic) and LNCaP-LN3 (highly metastatic)], in an effort to understand the biochemical basis of prostate cancer progression and to identify candidate biomarkers of progression 27. In addition, we hypothesised that DCA would be most effective in altering the metabolic profile of LNCaP-LN3 cells, as their glycolytic enzyme profile suggests a dependence on pyruvate to lactate metabolism 17.

## METHODS

### Cell lines and culture

The human prostate cancer cell line LNCaP was purchased from the American Type Culture Collection (ATCC) (http://www.lgcstandards-atcc.org/). The human LNCaP-LN3 prostate cancer cell line was a kind gift from Dr Curtis Pettaway (MD Anderson Cancer Center, Houston, TX, USA) 28.

Both cell lines were cultured in Dulbecco's modified Eagle's medium (DMEM) (high glucose + GlutaMAX) supplemented with 10% fetal calf serum (FCS) and 1 × penicillin/streptomycin (P/S) (Life Technologies, Paisley, Scotland, UK). Cells were cultured in T175cm^2^ flasks, incubated at 37 °C with a mixture of 5% CO_2_–95% air, and passaged routinely when they reached approximately 80–90% confluence.

### DCA treatment of cells

A final concentration of 50 mm DCA was used in the culture medium above, and the cells were exposed for 24 h.

### Growth rates

Growth rates were measured for both the LNCaP and LNCaP-LN3 cells over 4 days under standard growth conditions as described above. Around 1 × 10^4^ cells were seeded into each well of a 24-well plate and counted using a haemocytometer under a microscope (× 10 objective), as described below.

### LDH zymography assays

Zymography for LDH isoenzymes was performed using cellulose acetate membranes (Titan III, 94 × 76 mm^2^, Helena Biosciences, Gateshead, Tyne and Wear, UK) with Tris-glycine buffer according to the supplied manufacturer's instructions and as described previously 28–30. Following electrophoresis, the gels were placed in 10 mL of staining solution containing 5 mm NAD, 50 mm lithium lactate, 0.1 mm Tris-HCl (pH 8.6), 0.2 mm phenazinmethosulphate (PMS), 2.0 mm nitrobluetetrazolium (NBT) and 0.8 mm 3-(4,5-dimethylthiazol-2-yl)-2,5-diphenyltetrazolium bromide (MTT), and the colour was allowed to develop (3–6 min) in the dark at 37°C. Assays were performed to confirm the LDH isoenzyme profiles in the prostate cancer cells, and to correlate these with our NMR findings.

### Assessment of the viability of cells

In order to assess the percentage viability, cells were stained using trypan blue and counted on a haemocytometer under a microscope (× 10 objective) to differentiate between nonviable (blue-stained) and viable (unstained) cells. The viability of cells both with and without DCA treatment was assessed by culturing cells in 12-well plates following 3 days of growth. Following 24 h of DCA treatment, cells were stained using trypan blue solution, and the numbers of live and dead cells were counted as described above. In addition, the viability of cells was measured before and at 1 h after entry into the MR-compatible bioreactor system to estimate the rate of cell death caused by the NMR procedure. Cells were pipetted from the bioreactor and stained with trypan blue solution, from which the numbers of dead and living cells were counted.

### Bioreactor system

**Figure 2:**
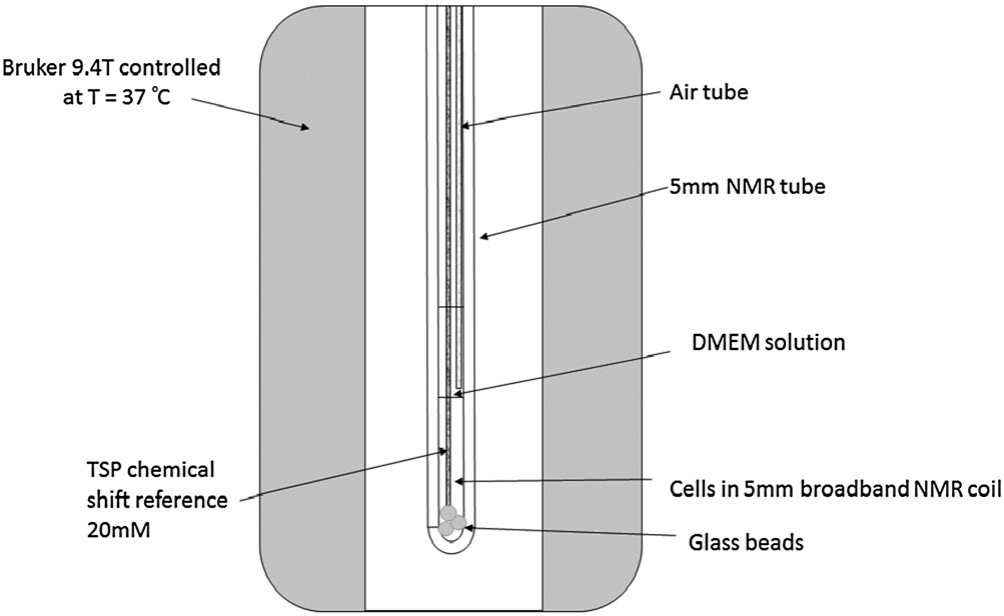
Schematic diagram of the bioreactor.

The design of the bioreactor was based on previously published designs 18,31,32, and consisted of a 5-mm-diameter NMR tube, which was initially filled to a depth of 20 mm with glass beads (Fig. 2) to ensure that cells were positioned in the sensitive region of the NMR coil 18. The phosphate-buffered saline-rinsed cell suspension of approximately 1 mL (which varied in cell number from 2.5 × 10^7^ to 1.1 × 10^8^ cells/mL) was then pipetted into the NMR tube. A 1-mm outer diameter coaxial tube containing 20 mm trimethylsilyl propionate (TSP) (dissolved in D_2_O) was inserted into the 5-mm NMR tube and used as a chemical shift reference; 2 mL of DMEM medium were pipetted on top of the cell suspension. A 0.96-mm outer diameter capillary tube (Smiths Medical, Ashford, Kent, UK), which provided air (21% oxygen) at a rate of 2.4 mL/min via a diaphragm pump, was then fixed on the top of the 5-mm NMR tube and placed with the exit approximately 10 mm above the top of the cell suspension. Temperature was controlled by the 9.4-T Bruker Avance III NMR Spectrometer (Bruker Biospin MRI GmbH, Ettlingen, Germany), set at 310 K and allowed to stabilise for 30 min prior to loading the bioreactor system.

### NMR data acquisition and analysis

All spectra were acquired using a Bruker 5-mm BBO probe. Water signal suppression was achieved using the Watergate suppression sequence (NUC1 = ^1^H; NS = 64; DS = 2; spectral width, 11 160 Hz; AQ = 0.2 s; D1 = 2 s; total time for spectral acquisition, 148 s). The Watergate zggpw5 sequence uses a hard 90° excitation pulse, followed by two composite radiofrequency pulses comprising five symmetrical pairs of pulses with different flip angles. Each composite pulse is surrounded by a pair of dephasing/rephasing gradients. The effect of the sequence is to effectively eliminate the water peak, whilst uniformly exciting the rest of the required spectrum. The flat excitation bandwidth of the sequence is determined by the spacing of the composite pulses and the acquisition window. Bruker Topspin 2.1 software was used for Fourier transform, automatic phasing, baseline correction to help remove broad baseline signals from macromolecules, line broadening (1 Hz) and zero filling. Chemical shifts were referenced by setting the TSP peak to 0 ppm or the lactate peak chemical shift confirmed by two-dimensional double quantum-filtered COrrelated SpectroscopY (DQF-COSY) and ^1^H–^13^C *heteronuclear single quantum coherence* (HSQC) assignment, which showed two peaks at 1.3 and 4.11 ppm. Spectral regions of interest were identified and then integrated automatically using Bruker TopSpin software.

### Metabolite ratio calculations and analysis

Major metabolite spectral regions were identified from ^1^H–^1^H DQF-COSY and ^1^H–^13^C HSQC spectra against the Spectral Database for Organic Compounds (SDBS, *sdbs.riodb.aist.go.jp*) and quantified. Lactate, alanine creatine + phosphocreatine and choline + glycerophosphocholine were used to form metabolite ratios. The integration limits were assigned as follows: lactate, 1.25–1.35 ppm; alanine, 1.35–1.5 ppm; creatine + phosphocreatine, 2.95–3.05 ppm; choline + glycerophosphocholine, 3.15–3.3 ppm. The assigned compound names are nominal and the integral regions also contain unidentified peaks. Metabolite ratios for lactate/creatine (Lac/Cr), lactate/choline (Lac/Cho) and choline/creatine (Cho/Cr) were calculated and analysed statistically using SPSS 20 (IBM, London, UK). In addition, a combined metabolite ratio marker comprising lactate/(choline + creatine + alanine) [Lac/(Cho + Cr + Ala)] was compared between cell types, with and without treatment. The TSP chemical shift reference peak at 0 ppm is also shown, although this was not used as a concentration standard because of inconsistent radiofrequency excitation by the Watergate pulse at this frequency, which was near to the second null of the excitation profile. The glucose region, glutamate/glutamine and leucine, isoleucine and valine regions of the spectrum were not included in the analysis because of possible interference from the DMEM solution required to maintain cell viability (see Discussion).

### Statistical analysis

An independent samples, Mann–Whitney, nonparametric, single-sided *U*-test was used to assess statistical significance at a level of *p* < 0.05 between cell types and treatment as the data were non-normally distributed. For the combined metabolite ratio marker, a multiple comparison correction was not required. For cell viability, data were also non-normally distributed, and thus Kruskal–Wallis and Mann–Whitney tests were used to compare differences, also at a significance level of *p* < 0.05, applying a Bonferroni correction for the number of tests.

## RESULTS

### Cell viability

**Figure 3:**
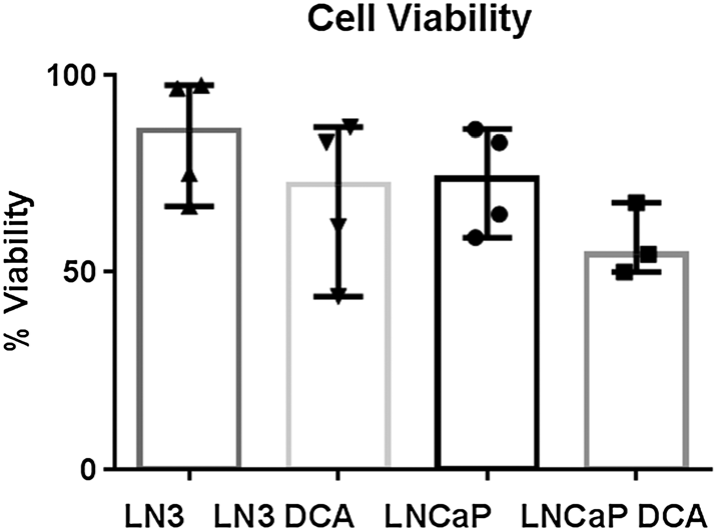
Plot showing the median and range of viability of cells by cell type and treatment prior to entry into the bioreactor. Each point represents an individual experiment. The untreated LNCaP-LN3 and LNCaP cells showed similar maximum viability values. A Kruskal–Wallis test showed that there was no significant difference in viability between the four treatment groups (*p* > 0.5).

Similar growth rates for LNCaP-LN3 and LNCaP cells were found over 4 days. Figure 3 shows that the viability of cells tended to decrease slightly following DCA treatment for both LNCaP and LNCaP-LN3 cells; however, this was found not to be statistically significant between the four groups (*p* > 0.5). In addition, the viability of cells measured after 1 h in the bioreactor system showed that the mean cell death across the groups was 2 ± 1%.

### Effect of DCA on metabolite ratios

**Figure 4:**
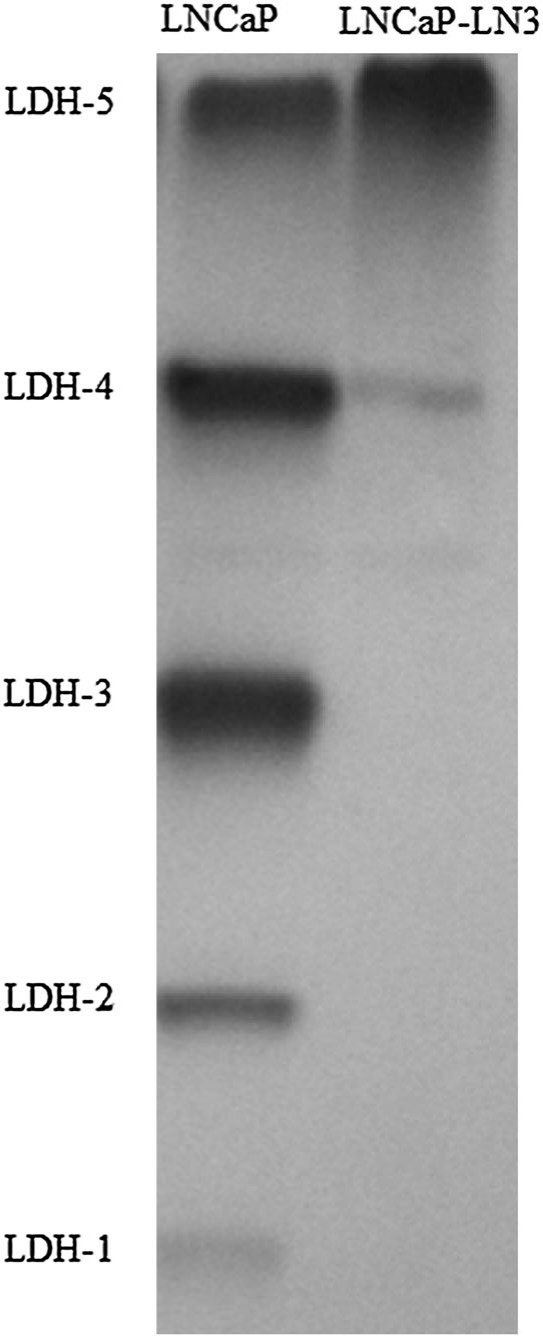
Lactate dehydrogenase (LDH) isoenzyme assay of the LNCaP and LNCaP-LN3 cell lines. A representative example from one of three experiments is shown. It should be noted that the LDH-5 isoenzyme is predominantly expressed in the LNCaP-LN3 cells, whereas all five isoenzymes (LDH-1–5) are expressed in the LNCaP cells. Equal amounts of protein were loaded for both cell lines.

**Figure 5:**
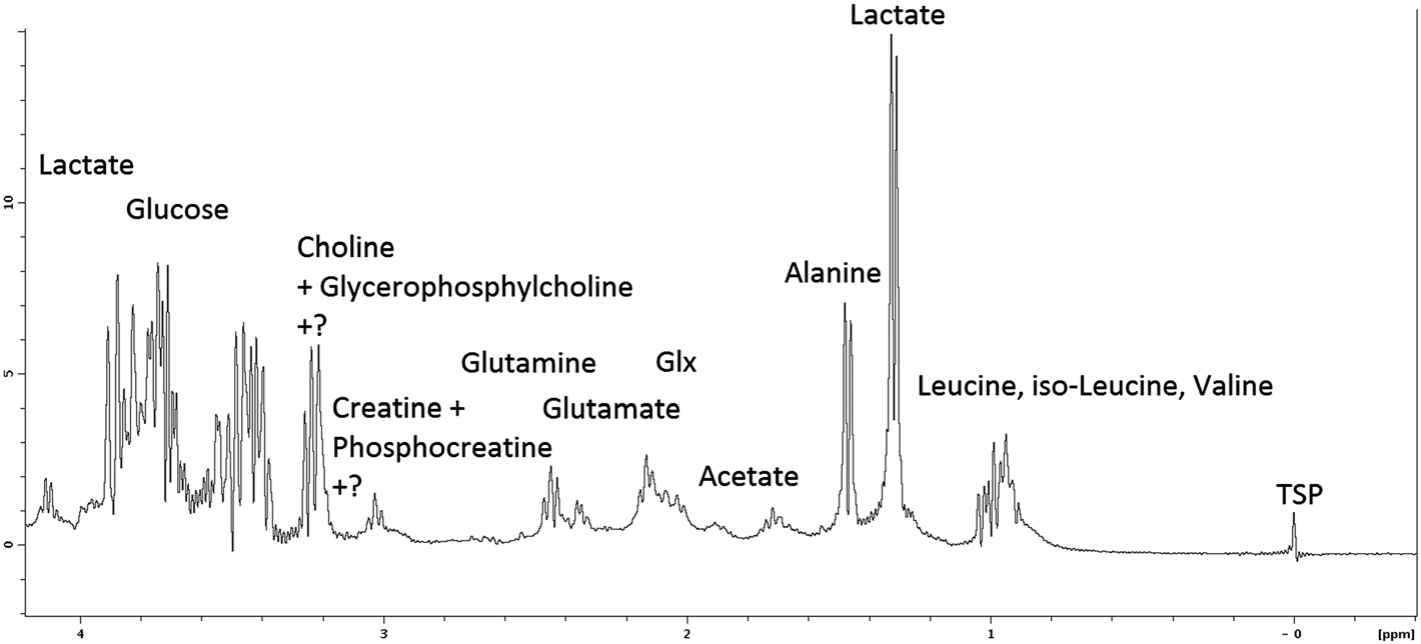
Example of representative high-resolution 1H spectrum from LNCaP-LN3 cells following 24 h of treatment with 50mM dichloroacetate (DCA) with nominal assignment labels. Some regions contain unidentified peaks. TSP, trimethylsilyl propionate.

Spectral peaks, identified from the ^1^H spectrum shown in Fig. 4, were integrated to produce metabolite Lac/Cr, Lac/Cho and Lac/Ala ratios. For LNCaP-LN3 cells, Lac/Cr, Lac/Cho and Lac/Ala were all significantly different following treatment (data not shown). In addition, the change in the combined metabolite ratio marker Lac/(Cho + Cr + Ala) also reached significance for LNCaP-LN3 cells following treatment. No metabolite ratios were significantly different following treatment of the LNCaP cells. There were also no significant differences for untreated LNCaP-LN3 and LNCaP cells. Figure 5 shows the Lac/(Cho + Cr + Ala) metabolite ratios with median (column) and 95% confidence intervals (bars) plotted for the LNCaP-LN3 and LNCaP cells, before and after treatment with 50 mm DCA.

### LDH zymography

**Figure 6:**
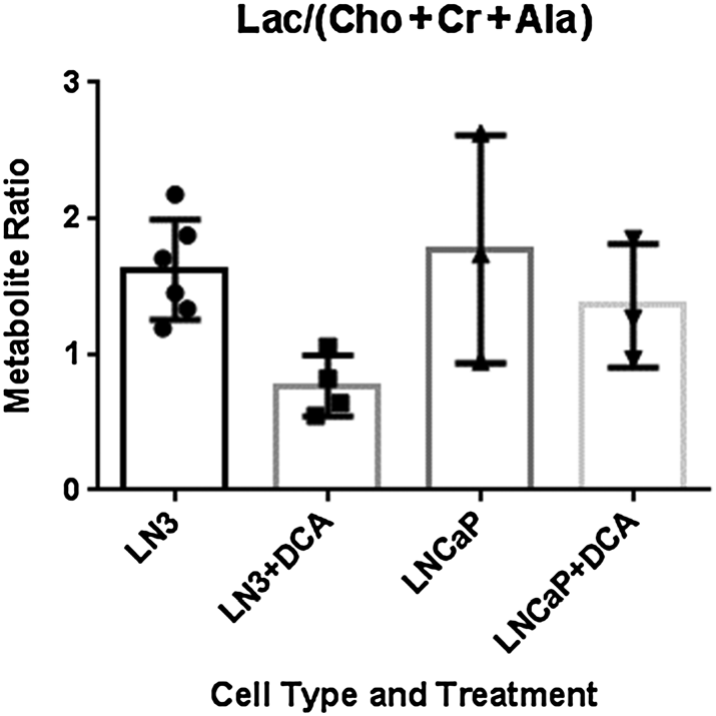
Combined metabolite ratio marker [lactate/(choline+ creatine+ alanine), Lac/(Cho+Cr+ Ala)] for LNCaP-LN3 cells pre- (n =6) and post-treatment (n = 4) and LNCaP cells pre- (n = 3) and post-treatment (n = 3) with 50mM dichloroacetate (DCA) for 24 h before measurement. Columns represent median ratios ± 95% confidence intervals. The only significant difference in the nonparametric Mann–Whitney U-test (p<0.05) was between pre- and post-treatment with DCA of LNCaP-LN3 cells.

The LDH isoenzyme expression in LNCaP-LN3 and LNCaP cancer cell lines is shown in Fig. 6. Expression of all five LDH isoenzymes (LDH-1, LDH-2, LDH-3, LDH-4 and LDH-5) can be seen in LNCaP cells, whereas absent or reduced expression of isoenzymes LDH-1, LDH-2, LDH-3 and LDH-4 can be seen in LNCaP-LN3 cells. These results confirm previous findings for these cell lines 29.

## DISCUSSION

Our results demonstrate similar levels of lactate/metabolite ratios in two untreated prostate cancer cell lines with very different metastatic potentials. In contrast, there were differences in the response of the cell lines to DCA, with several of the measured lactate/metabolite ratios significantly decreased after DCA treatment in the highly metastatic LNCaP-LN3 cells, but not in the poorly metastatic LNCaP cells. These differences may relate to a higher prevalence of LDH-A subunits in the LDH isoenzyme, seen in LNCaP-LN3 relative to LNCaP cells.

Following 1 h after cell entry into the bioreactor system, viability was assessed and a mean cell death across groups of 2 ± 1% was noted. This meant that cells were still alive during data acquisition, and therefore our data can be used for the measurement and interpretation of active cell metabolite ratios.

Some spectral interference from diffusion of the support medium DMEM into the active MR coil volume containing the cells, required to maintain cell viability, would be expected. The partial volume of DMEM in the active coil region during acquisition has been estimated to be 10% of the cell volume using the known concentration of glucose of 25 mm in DMEM and the volume and concentration (20 mm) of TSP contained within the tube. DMEM contains many of the same metabolic substances as found in cells, but most of the relevant amino acids and support compounds are at concentrations much less than 1 mm (www.sigma-aldrich.com). Therefore, because of the partial volume factor, they would not contaminate the measured spectra significantly. An exception is d-glucose, which is present in DMEM at a very high concentration of 25 mm. In terms of amino acids, l-glutamine is the other high-concentration constituent of DMEM at 4 mm, and leucine, isoleucine and valine are present at 0.8 mm. For this reason, the glucose, glutamine and leucine, isoleucine and valine regions of the spectra were not included in the analysis. All the other compounds in DMEM are at very low concentrations and so would not be expected to affect the measured spectra significantly.

As expected, the LNCaP-LN3 cells principally showed the expression of the LDH-5 (LDH-A4) isoenzyme, with relatively reduced/absent expression of isoenzymes 1–4 (Fig. 6). This finding is consistent with the absent or reduced expression of the LDH-B subunit in LNCaP-LN3 cells, as reported previously 29. Loss of the LDH-B subunit in prostate, breast and gastric cancers has been reported to involve promoter hypermethylation 29,30,33. The remaining LDH-5 isoenzyme is composed solely of four LDH-A subunits (LDH-A4) 10,34. Thus, considering the very different LDH isoenzyme profiles of our cell lines, our finding of no significant differences in lactate/metabolite ratios between the highly metastatic LNCaP-LN3 cells and the poorly metastatic LNCaP cells was somewhat unexpected. A possible explanation for this is that the LNCaP cells rely heavily on processes such as glutamine metabolism to generate lactate 12,13. It is also possible that any differences in lactate/metabolite ratios between the LNCaP and LNCaP-LN3 cells would only become evident following hypoxic growth conditions, as shown recently using breast cancer cell lines that lack the LDH-B subunit 29.

Although a concentration standard (20 mm TSP) was included within the acquisition volume, absolute concentrations were not used in the analysis because the Watergate water suppression sequence second excitation null partially suppressed the 0-ppm region of the spectrum. For this reason, we used TSP purely as a chemical shift reference.

Following DCA treatment, the Lac/Cr, Lac/Cho and Lac/Al ratios all decreased significantly in the highly metastatic LNCaP-LN3 cells. Cho/Cr was not changed significantly using the same test. The combined metabolite ratio marker Lac/(Cho + Cr + Ala) was also significantly different (*p* < 0.05). Using the combined marker removes the need for a multiple comparison correction. Conversely, no changes in metabolite ratios were noted following DCA treatment of LNCaP cells. Increased sensitivity of LNCaP-LN3 relative to LNCaP cells may relate to their LDH isoenzyme profile, which suggests that they rely heavily on lactate production for their survival and proliferation. Clearly, further studies are required to substantiate the negative effect of DCA on LNCaP cells and to investigate the exact mechanism(s) underlying the differences between the two cell types.

DCA treatment is expected, from other studies, to lead to an increased flux through the tricarboxylic acid (TCA) cycle and to induce oxidative stress 35. It has been shown that DCA can act to facilitate apoptosis of prostate cancer cells 27, and a previous study has demonstrated 25% inhibition of cell growth following 96 h of 1 mm and 0.5 mm DCA in two prostate cancer cell lines, PC-3-Bcl-2 and PC-3-Neo. Another study, which used hypoxic HeLA cervical and PANC-1 pancreatic cancer cells to evaluate *in vitro* effects of DCA, concluded that, following 24 h of treatment, 94% of the cells were viable at doses of 12.5 mm, whereas, at 96 h of treatment, only 64% were viable 36. Furthermore, in HT29 and LoVo cell lines isolated from colorectal adenocarcinomas, there were increases of 2.8% and 21%, respectively, in total apoptotic cells following 50 mm of DCA treatment for 48 h 37. Based on these reported studies, DCA toxicity requires significant time to manifest, and this could explain why we only saw a tendency towards decreased viability after 24 h of treatment in our cell lines. Further studies are required to determine whether longer exposures to DCA would induce selective toxicity in the metabolically sensitive LNCaP-LN3 cell line.

The importance of lactate in tumour progression, including in prostate cancer development, has been established in many studies. For instance, lactate has been associated with a low extracellular pH in tumours, which can increase extracellular matrix breakdown and aid in the regulation of angiogenic factors, such as vascular endothelial growth factor (VEGF) and interleukin-8 38. In addition, human studies have shown a strong correlation between tumour lactate concentration following biopsy and the incidence of metastasis 39.

These considerations have led to a major worldwide research effort into the targeting of the abnormal metabolism of tumours for therapeutic benefit. Treatment with doses of DCA as high as 125 mg/kg/day have been used in humans for conditions such as lactic acidosis, although some studies have reported peripheral nerve effects with doses as low as 25 mg/kg/day. DCA mainly has an effect on cell metabolism, enhancing the entry of pyruvate into the TCA cycle through repolarisation of the cell membrane, rather than reducing cell viability, i.e. it is not specifically cytotoxic (www.thedcasite.com/dca_human_studies.html). Although DCA may prove to be unsuitable for further clinical development, other routes for the inhibition of PDK are actively underway 40,41. Numerous preclinical studies with DCA have shown a variable response between different tumour models, highlighting the need for predictive biomarkers of efficacy 25–27. Our results suggest that further investigation of the prevalence of LDH-A subunits in the LDH isoenzyme of tumour cells, for predicting the response to PDK inhibitors, is warranted. Despite the fact that the LDH-A subunit predominates in LDH of LNCaP-LN3 cells, no differences were found in the metabolic profiling of these cells *versus* the LNCaP cells, although a difference in response to the effects of DCA was observed for the highly metastasising cells.

## CONCLUSIONS

The highly metastatic LNCaP-LN3 cells appeared to respond metabolically with an inferred decreased production of lactate when treated with DCA, whereas the poorly metastatic LNCaP cells did not. The LDH isoenzyme profile appears to be significant in controlling the response of cells to the effects of treatment with DCA. These findings are of importance, given that previous studies 29 have shown an absence of the LDH-B subunit to be a common feature of both human prostate and breast cancer cases 30.

## Abbreviations

*AQ*: *Acquisition time*
*Cho/Cr*: *choline/creatine*
*DCA*: *dichloroacetate*
*D1*: *Relaxation delay*
*DMEM*: *Dulbecco's modified Eagle's medium*
*DQF-COSY*: *double quantum-filtered correlation spectroscopy*
*DRE*: *digital rectal examination*
*DS*: *Number of steady state scans*
*FCS*: *fetal calf serum*
*HSQC*: *heteronuclear single quantum correlation*
*Lac/Ala*: *lactate/alanine*
*Lac/Cho*: *lactate/choline*
*Lac/Cr*: *lactate/creatine*
*Lac/(Cho + Cr + Ala)*: *lactate/(choline + creatine + alanine)*
*LDH*: *lactate dehydrogenase*
*MTT*: *3-(4,5-dimethylthiazol-2-yl)-2,5-diphenyltetrazolium bromide*
*NAD^+^/NADH*: *oxidised/reduced forms of nicotinamide adenine dinucleotide*
*NBT*: *nitrobluetetrazolium*
*NS*: *Number of Scans*
*NUC1*: *Nucleus*
*PDK*: *pyruvate dehydrogenase kinase*
*PMS*: *phenazinmethosulphate*
*P/S*: *penicillin/streptomycin*
*PSA*: *prostate-specific antigen*
*SWH*: *Spectral width*
*TCA*: *tricarboxylic acid*
*TRUS*: *trans-rectal ultrasound*
*TSP*: *trimethylsilyl propionate (sodium salt)*
*VEGF*: *vascular endothelial growth factor.*
